# Supplementary material for: Gut Microbiota Bacterial Species Associated with Mediterranean Diet-Related Food Groups in a Northern Spanish Population
Source: Nutrients. 2021 Feb 16;13(2):636. doi: 10.3390/nu13020636 (PMC7920039; doi:10.3390/nu13020636)
Supplement: Supplementary file 1 [file nutrients-13-00636-s001.pdf]

Figure S1. NMDS (Non-metric Multidimensional Scaling) graph comparing the beta diversity of the tertiles with higher (blue) and lower (red) adherence to MD.

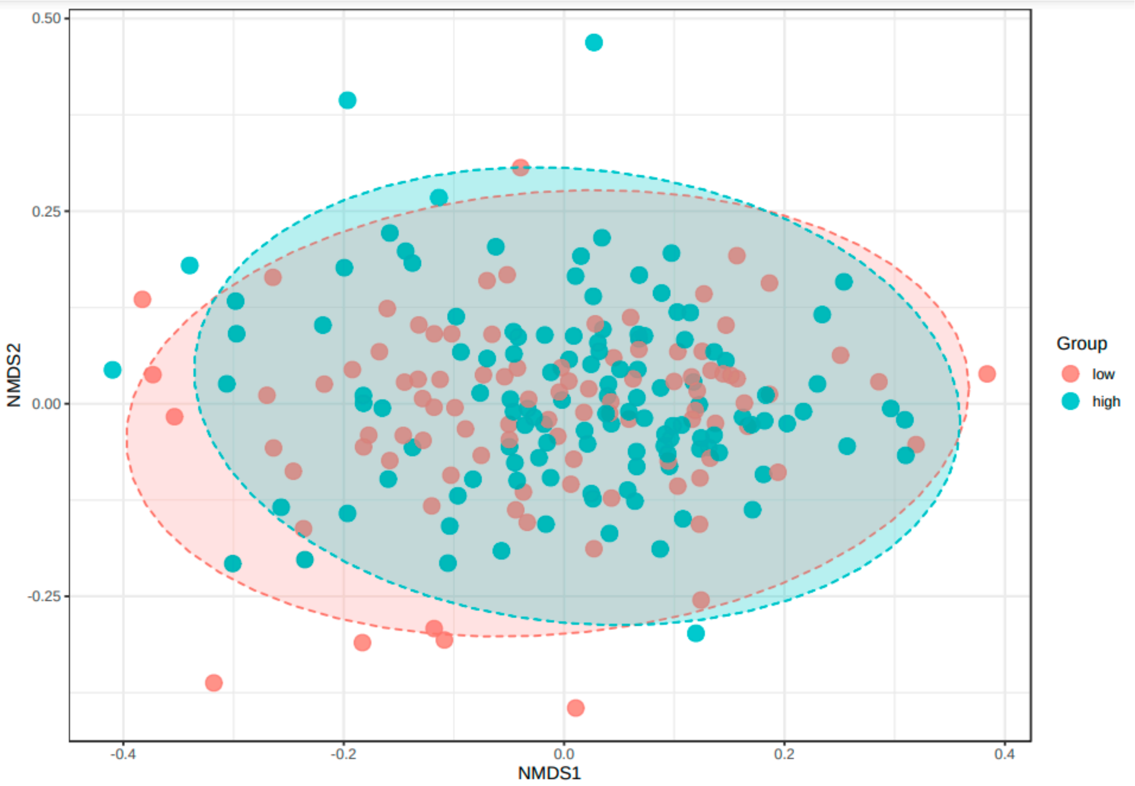

Table S1. Baseline characteristics of the entire population separated by sex and weight status.

Variables	Women (n = 251)	Men (n = 109)	Normal Weight (n = 64)	Overweight + Obese (n = 110+186)
Age (y)	44.6 ± 0.6	45.3 ± 0.9	39.8 ± 1.1	45.9 ± 0.6
Weight (kg)	78.8 ± 0.9	92.9 ±1.3	61.7 ± 1.2	87.7 ± 0.7
BMI	29.8 ± 0.3	30.2 ± 0.4	22.1 ± 0.2	31.6 ± 0.2
Waist circumference (cm)	94.7 ± 0.9	103.8 ± 1.2	75.6 ± 0.9	102.2 ± 0.6
Hip circumference (cm)	109.9 ± 0.6	105.9 ± 0.7	94.7 ± 0.7	111.8 ± 0.5
Glucose (mg/dL)	93 ± 1	99 ± 1	85 ± 1	97 ± 1
Total cholesterol (mg/dL)	211 ± 2	216 ± 4	193 ± 4	216 ± 2
HDL-c (mg/dL)	60 ± 1	49 ± 1	63 ± 1	55 ± 1
LDL-c (mg/dL)	132 ± 2	144 ± 3	116 ± 4	140 ± 2
Triglycerides (mg/dL)	89 ± 3	116 ± 7	68 ± 4	104 ± 3
HOMA-IR	1.6 ± 0.1	1.9 ± 0.1	0.9 ± 0.1	1.9 ± 0.1
Carbohydrate intake (%)	41.2 ± 0.4	41.0 ± 0.6	43.5 ± 0.8	40.6 ± 0.4
Protein intake (%)	17.3 ± 0.2	16.0 ± 0.2	16.8 ± 0.4	17.0 ± 0.2
Fat intake (%)	40.2 ± 0.4	39.7 ± 0.5	38.1 ± 0.7	40.5 ± 0.3
Fiber intake	28.9 ± 11.5	27.8 ±11.1	31.3 ± 14.6	28.0 ± 10.6
Energy intake (kcal/day)	2796 ± 55	3165 ± 92	2618 ± 88	2969 ± 55

Values correspond to the mean ± SEM (Standard Error of the Mean). HDL: High-density lipoprotein cholesterol; LDL: Low-density lipoprotein cholesterol; HOMA-IR (The homeostatic model assessment for insulin resistance): insulin resistance index.
